# Draft Genome Sequence of a Medium- and Long-Chain *n*-Alkane-Degrading Bacterium, Tsukamurella tyrosinosolvens Strain PS2, with Two Genetic Systems for Alkane Degradation

**DOI:** 10.1128/MRA.00218-19

**Published:** 2019-04-25

**Authors:** Valeriya A. Romanova, Eugenia A. Boulygina, Maria N. Siniagina, Maria I. Markelova, Tatiana V. Grigoryeva, Alexander V. Laikov

**Affiliations:** a Institute of Fundamental Medicine and Biology, Kazan Federal University, Kazan, Russia; Broad Institute of MIT and Harvard University

## Abstract

Here, we report the genome sequence of Tsukamurella tyrosinosolvens strain PS2, which was isolated from hydrocarbon sludge of an organic synthesis factory. This strain was able to utilize a wide range of *n*-alkanes, from C_16_ to C_35_, as sole carbon sources. Knowledge of the genome will provide insights into long-chain *n*-alkane biodegradation mechanisms.

## Abstract (precis)

Here, we report the genome sequence of Tsukamurella tyrosinosolvens strain PS2, which was isolated from hydrocarbon sludge of an organic synthesis factory. This strain was able to utilize a wide range of *n*-alkanes, from C_16_ to C_35_, as sole carbon sources.

## ANNOUNCEMENT

The species Tsukamurella tyrosinosolvens was first isolated from blood cultures of humans with cardiac pacemaker implants (1), and it had been considered a conditional pathogen (2–5). In 2016, reclassification of species within the genus Tsukamurella was performed, and the species T. tyrosinosolvens and Tsukamurella carboxydivorans were merged (6). Thus, a wider variety of habitats, including soil and oil-polluted areas, were assigned to the species *T. tyrosinosolvens* (7, 8).

A bacterial strain, *T. tyrosinosolvens* PS2, was isolated from chemical sludge with high hydrocarbon contamination (up to 350 g/kg of bichromate-oxidized matter) that had been stored for 10 years in a sludge repository (9). This strain was able to grow on hexadecane and solid paraffin as the sole carbon and energy sources (10). In order to provide a better understanding of extreme contamination resistance and long-chain hydrocarbon degradation mechanisms, *T. tyrosinosolvens* PS2 genome sequencing was performed.

Genomic DNA was extracted from pure culture grown on LB medium according to a miniprep protocol, with detergent lyses and phenol-chloroform purification performed as described by Wilson (11). The quantity of extracted DNA was measured with a Qubit 2.0 fluorometer (Invitrogen). After fragmentation of genomic DNA by a Covaris ultrasonication system, the DNA library was constructed with a NEBNext Ultra library preparation kit, according to the manufacturer’s instructions. The quality of the resulting library was estimated using an Agilent 2100 Bioanalyzer. Sequencing was performed on an Illumina MiSeq platform using a v3 reagent kit and following the manufacturer’s protocol. Paired-end (2 × 300-bp) sequencing generated a total of 10,773,156 raw reads. After adapter removal and filtering by length and quality using Cutadapt (12) (flags –m 20 –M300 –q 20), 5,473,362 paired-end reads were used for the genome *de novo* assembly using SPAdes v3.1.1 (13). The draft genome contains 20 contigs (the first 6 contigs covering over 4.8 Mbp), the genome coverage is 245×, and the GC content is 71.32%. The total assembly length is 4,845,031 bp, and the *N*_50_ value is 1,229,895 bp. Genome annotation performed using Prokka v1.7 (14) and CRISPRCasFinder (15) resulted in 4,760 protein-coding sequences, 53 tRNA-coding genes, and 5 CRISPR cassettes. Based on PlasmidFinder v2.0 (16) search results, plasmid DNA was not detected.

In contrast to the previously described representatives of the genus *Tsukamurella* with respect to hydrocarbon biodegradation, two alkane catabolic pathways were found in the *T. tyrosinosolvens* PS2 genome. The first pathway contains an alkane 1-monooxygenase gene (*alkB*; GenBank accession number KZL97795), two rubredoxin genes (*rubA*; GenBank accession numbers KZL97794 and KZL97793), one rubredoxin-reductase gene (*rubB*), and one regulatory protein TetR gene (*alkU*; GenBank accession number KZL97792). This pathway is similar to that from the alkane-degrading actinomycete *T. tyrosinosolvens* strain MH1, as follows from the complete genome (8). Also a homologous gene of cytochrome P450 alkane 1-monooxygenase of the Gordonia sp. strain TF6 (GenBank accession number BAF95905), which is known to play a key role in alkane degradation, was found in the *T. tyrosinosolvens* PS2 genome (GenBank accession number KZL95198). Thus, two detected systems may benefit the strain in an extremely polluted environment and will provide insights into the ecological role of this bacterium.

### Data availability.

The whole-genome sequence reported here has been deposited at GenBank under the accession number LSFR00000000. The BioProject accession number is PRJNA311144. Raw data have been deposited in the SRA under the accession number SRX2363910.
